# Therapeutic activity of a combination of immunostimulatory monoclonal antibodies (anti-B7-H1, CD137 and OX40) on a c-myc-driven spontaneous transgenic model of hepatocellular carcinoma

**DOI:** 10.1186/2051-1426-1-S1-O7

**Published:** 2013-11-07

**Authors:** Aizea Morales-Kastresana, Sanmamed F Miguel, Inmaculada Rodriguez, Asis Palazon, Ivan Martinez-Forero, Sara Labiano, Sandra Hervas-Stubbs, Bruno Sangro, Carmen Ochoa, Ana Rouzaut, Arantza Azpilikueta, Elixabet Bolaños, Maria Jure-Kunkel, Ines Gutgemann, Ignacio Melero

**Affiliations:** 1 CIMA, Pamplona, Spain; 2 CUN, Pamplona, Spain; 3 Bristol Myers Squibb, Lawrenceville, NJ, USA; 4 University of Bonn, Bonn, Germany

## 

Several immunostimulatory monoclonal antibodies (ISmAbs) that derepress and unleash antitumor immune responses are showing efficacy in cancer clinical trials. MAbs anti-B7-H1 (PD-L1) block a critical inhibitory pathway for T cells, while antibodies anti-CD137 and OX40 provide intense T cell costimulation. Documenting efficacy of immunotherapies on spontaneous tumors arising in oncogene transgenic mice is considered more predictive than experiments on transplanted tumors. A combination of these ISmAbs has been tested on a transgenic mouse model of spontaneous primary liver cancer in which c-myc drives transformation and the tumor cells express ovalbumin as a surrogate transgenic antigen. The induced tumor lymphocyte infiltrates and immune mechanisms of action were studied. The triple combination of mAbs clearly extended survival of mice bearing hepatocellular carcinomas (HCC) and synergized with adoptive T cell therapy with activated TCR-transgenic T cells that recognize OVA. Mice undergoing therapy showed a clear increase in the tumor tissue infiltration by activated and blastic CD8 T lymphocytes expressing the ISmAb-targeted receptors. The triple combination of ISmAbs did not result in enhanced OVA-specific CTL activity but other antigens in cell lines derived from such HCC were recognized by the elicited tumor infiltrating T lymphocytes. Indeed adoptive transfer of OT-1 cells to tumor bearing mice resulted in their tolerization, unless the triple mAb therapy was instigated. Our results emphasize the role of combinational immunotherapy approaches including ISmAbs to impact on agressive and highly T cell-tolerizing hepatocellular carcinomas.

